# Using Natural Language Processing (GPT-4) for Computed Tomography Image Analysis of Cerebral Hemorrhages in Radiology: Retrospective Analysis

**DOI:** 10.2196/58741

**Published:** 2024-09-26

**Authors:** Daiwen Zhang, Zixuan Ma, Ru Gong, Liangliang Lian, Yanzhuo Li, Zhenghui He, Yuhan Han, Jiyuan Hui, Jialin Huang, Jiyao Jiang, Weiji Weng, Junfeng Feng

**Affiliations:** 1 Brain Injury Centre Ren Ji Hospital Shanghai Jiao Tong University School of Medicine Shanghai China; 2 Shanghai Institute of Head Trauma Shanghai China; 3 Department of Radiology Ren Ji Hospital Shanghai Jiao Tong University School of Medicine Shanghai China; 4 Department of Radiology Minhang Hospital Fudan University Shanghai China

**Keywords:** GPT-4, natural language processing, NLP, artificial intelligence, AI, cerebral hemorrhage, computed tomography, CT

## Abstract

**Background:**

Cerebral hemorrhage is a critical medical condition that necessitates a rapid and precise diagnosis for timely medical intervention, including emergency operation. Computed tomography (CT) is essential for identifying cerebral hemorrhage, but its effectiveness is limited by the availability of experienced radiologists, especially in resource-constrained regions or when shorthanded during holidays or at night. Despite advancements in artificial intelligence–driven diagnostic tools, most require technical expertise. This poses a challenge for widespread adoption in radiological imaging. The introduction of advanced natural language processing (NLP) models such as GPT-4, which can annotate and analyze images without extensive algorithmic training, offers a potential solution.

**Objective:**

This study investigates GPT-4’s capability to identify and annotate cerebral hemorrhages in cranial CT scans. It represents a novel application of NLP models in radiological imaging.

**Methods:**

In this retrospective analysis, we collected 208 CT scans with 6 types of cerebral hemorrhages at Ren Ji Hospital, Shanghai Jiao Tong University School of Medicine, between January and September 2023. All CT images were mixed together and sequentially numbered, so each CT image had its own corresponding number. A random sequence from 1 to 208 was generated, and all CT images were inputted into GPT-4 for analysis in the order of the random sequence. The outputs were subsequently examined using Photoshop and evaluated by experienced radiologists on a 4-point scale to assess identification completeness, accuracy, and success.

**Results:**

The overall identification completeness percentage for the 6 types of cerebral hemorrhages was 72.6% (SD 18.6%). Specifically, GPT-4 achieved higher identification completeness in epidural and intraparenchymal hemorrhages (89.0%, SD 19.1% and 86.9%, SD 17.7%, respectively), yet its identification completeness percentage in chronic subdural hemorrhages was very low (37.3%, SD 37.5%). The misidentification percentages for complex hemorrhages (54.0%, SD 28.0%), epidural hemorrhages (50.2%, SD 22.7%), and subarachnoid hemorrhages (50.5%, SD 29.2%) were relatively high, whereas they were relatively low for acute subdural hemorrhages (32.6%, SD 26.3%), chronic subdural hemorrhages (40.3%, SD 27.2%), and intraparenchymal hemorrhages (26.2%, SD 23.8%). The identification completeness percentages in both massive and minor bleeding showed no significant difference (*P*=.06). However, the misidentification percentage in recognizing massive bleeding was significantly lower than that for minor bleeding (*P*=.04). The identification completeness percentages and misidentification percentages for cerebral hemorrhages at different locations showed no significant differences (all *P*>.05). Lastly, radiologists showed relative acceptance regarding identification completeness (3.60, SD 0.54), accuracy (3.30, SD 0.65), and success (3.38, SD 0.64).

**Conclusions:**

GPT-4, a standout among NLP models, exhibits both promising capabilities and certain limitations in the realm of radiological imaging, particularly when it comes to identifying cerebral hemorrhages in CT scans. This opens up new directions and insights for the future development of NLP models in radiology.

**Trial Registration:**

ClinicalTrials.gov NCT06230419; https://clinicaltrials.gov/study/NCT06230419

## Introduction

Cerebral hemorrhage mainly encompasses intracranial bleeding resultant from trauma, hypertension, or cerebral vascular disorders. Typically, it manifests acutely, with severe symptoms and a prognosis that is often unfavorable, thereby imposing substantial economic burdens on individuals, families, and society [1-3]. Hence, the prompt and accurate diagnosis of cerebral hemorrhage is of paramount clinical and societal importance.

Computed tomography (CT) scanning stands as the quintessential diagnostic tool for cerebral hemorrhage. An expedient and precise diagnosis not only facilitates the comprehensive evaluation of patient conditions, the optimization of therapeutic strategies, and the prognostication of outcomes but also enhances communication between clinicians and patients or their respective families. Nonetheless, several challenges persist. First, CT diagnosis of cerebral hemorrhage often requires experienced radiologists. In low-income areas or countries, there is a significant shortage of these specialists [4,5]. Radiologists lacking sufficient experience will lead to missed or incorrect diagnosis, which can be potentially fatal in the clinical management of cerebral hemorrhage. Second, CT diagnosis of cerebral hemorrhage requires rapid reporting, but even in high-income areas and countries, the substantial workload makes it difficult for radiologists to provide prompt and comprehensive radiology reports [6]. Third, cultivating an experienced radiologist requires a mature medical education system and a substantial database of imaging resources, which is currently lacking in China [7].

Artificial intelligence (AI) has emerged as a formidable instrument in computer-aided diagnosis across various medical fields, enhancing classification [8], detection [9], and image segmentation [10]. AI proves particularly beneficial in supporting decision-making and rapid diagnosis, especially in underserved rural areas or densely populated regions with a scarcity of medical imaging experts [11]. Moreover, AI has substantially advanced medical education [12-14].

Currently, various AI models, including You Only Look Once and bespoke convolutional neural networks [15-17], have demonstrated efficacy in detecting cerebral hemorrhages in CT imagery. However, the construction and utilization of these AI models necessitate an understanding of computer algorithms. For most radiologists, the implementation of these models poses a significant challenge, indicating a need for more intuitive and accessible AI-driven diagnostic solutions.

The recent introduction of new natural language processing (NLP) models, such as ChatGPT (OpenAI) and the latest iteration of GPT-4, has enabled imaging analysis capabilities without the need for intricate algorithmic knowledge and extensive training [18,19]. Previously, large language models faced the challenge of hallucination, presenting presumably persuasive results that were not based on the input [20,21]. However, many studies suggest that GPT-4 can assist in generating, extracting, and interpreting radiology reports [22-24]. GPT-4 can play a role in the diagnosis and treatment of oral diseases, orthopedic diseases, neurological disorders, and pulmonary diseases using radiology reports [23,25-27]. GPT-4 is capable of accurately extracting lesion information from radiology reports, such as identifying metastatic diseases and generating correct labels for tumor progression [23,24]; it can integrate radiology reports and medical history to make diagnoses [27]; and it can even provide some treatment suggestions based on the radiology reports [26]. However, current research focuses only on GPT-4’s capabilities with free text, and its potential for imaging recognition has not yet been explored.

This work aims to delineate our study by utilizing GPT-4 for the identification and annotation of cerebral hemorrhages in CT images, including various types of intracranial bleeding. It is the first attempt to directly use GPT-4 to identify CT images of cerebral hemorrhages. It broadens the horizon for GPT-4 applications in the medical field, offering fresh perspectives for the development of GPT-4 and other NLP models in health care.

## Methods

### Ethical Considerations

This retrospective study was registered at ClinicalTrials.gov (NCT06230419) and approved by the Ethics Committee of Ren Ji Hospital, Shanghai Jiao Tong University School of Medicine (IIT-2024-0006). This study exclusively utilized CT images, which do not include personally identifiable information or sensitive individual data. There was no direct interaction with patients, complying with the principles of ethical conduct in research. Meanwhile, the Ethics Committee of Ren Ji Hospital, Shanghai Jiao Tong University School of Medicine, approved the usage of CT images without any identifiable private information. All the data are securely stored and only accessed by personnel involved in the research team.

### Study Design

**Figure 1:**
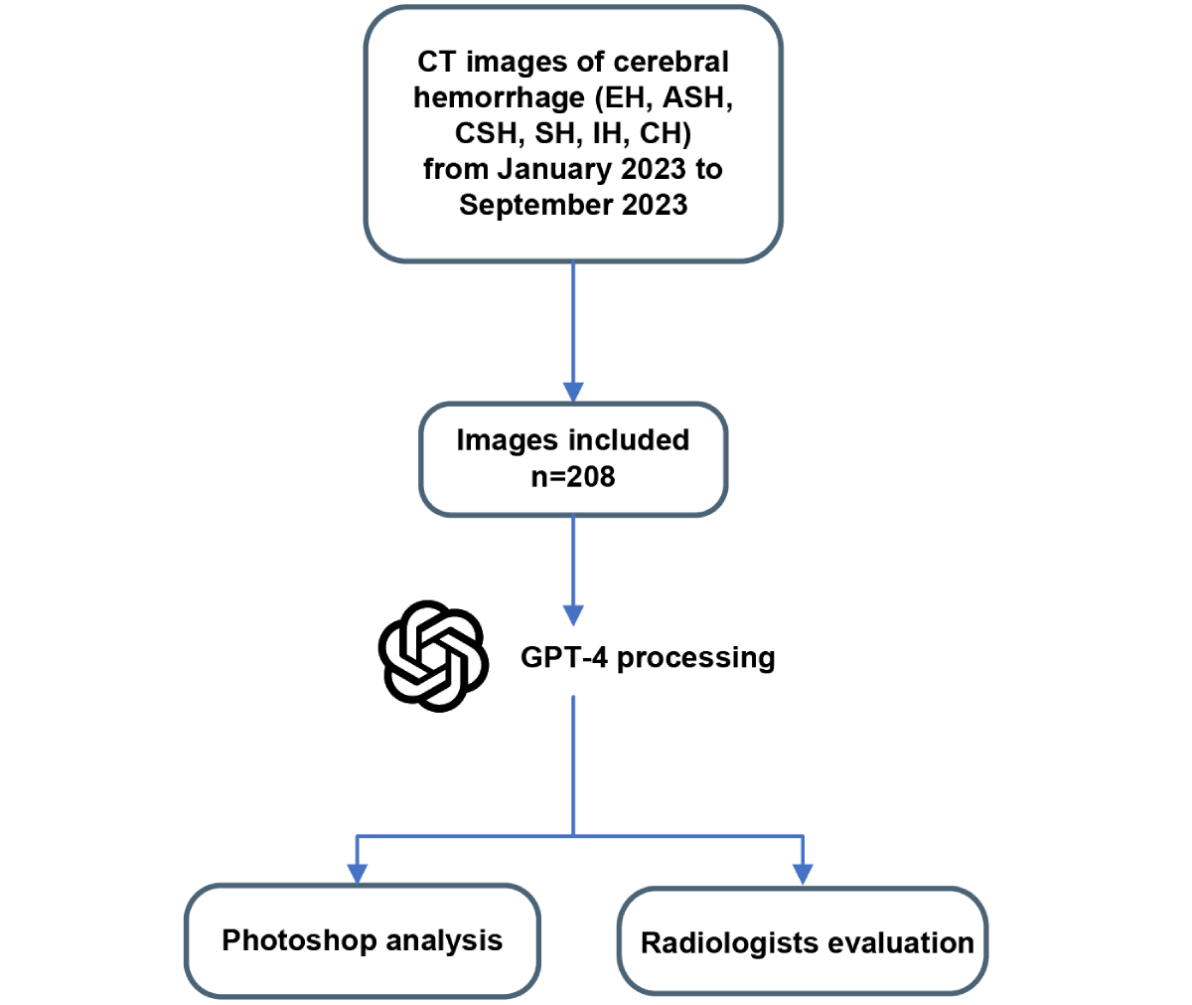
Flowchart of the study design. ASH: acute subdural hemorrhage; CH: complex hemorrhage; CSH: chronic subdural hemorrhage; CT: computed tomography; EH: epidural hemorrhage; IH: intraparenchymal hemorrhage; SH: subarachnoid hemorrhage.

As shown in Figure 1, raw CT images of different types of cerebral hemorrhages were collected between January and September 2023 from the radiology database of Ren Ji Hospital, Shanghai Jiao Tong University School of Medicine. Since GPT-4 cannot recognize continuous CT images, we first preprocessed the CT images. We chose the horizontal cranial CT image with the largest volume of hemorrhage in the brain window (window width: 90, window level: 35) as the representative image. Representative CT images were exported to be unified as JPG format files with a size of 700×700 pixels to minimize the influence from the image format and the image size.

By employing a predefined question-and-answer mode, we guided GPT-4 to annotate the hemorrhagic lesions in the provided images (Multimedia Appendix 1). All CT images of different types of cerebral hemorrhages were mixed together and each CT image was sequentially numbered, so all 208 CT images had their own corresponding number. A random sequence from 1 to 208 was generated using Python (version 3.8; Python Software Foundation), and all CT images were inputted into GPT-4 for analysis in the order of the random sequence. Once GPT-4 finished the analyze process, all annotated pictures were downloaded and saved as JPG format files.

All processed CT images were both analyzed by Photoshop (version 24.3.0; Adobe) and given to 10 radiologists to evaluate identification completeness, accuracy, and success.

### GPT-4 Update

We utilized the latest updated GPT-4 released by OpenAI in November 2023, which at present, empowers more powerful image analysis in GPT-4.

### Photoshop Analysis

After all the processed images were collected, the hemorrhage areas and the annotation areas were isolated from the images using Photoshop [28]. According to previous studies, the ABC/2 method has been universally applied in clinical medicine to estimate cerebral hemorrhage volumes swiftly [29,30]. The same method was used in the calculation of intraparenchymal hemorrhage volumes, given that intraparenchymal hemorrhages typically conform to the assumption of this formula: the hemorrhages approximate an elliptical shape. After tallying all intraparenchymal hemorrhage volumes, based on the corresponding bleeding areas measured using Photoshop, the hemorrhage volume per pixel was determined and the hemorrhage volume for each CT image in the other 5 types of irregular cerebral hemorrhages was calculated.

**Figure 2:**
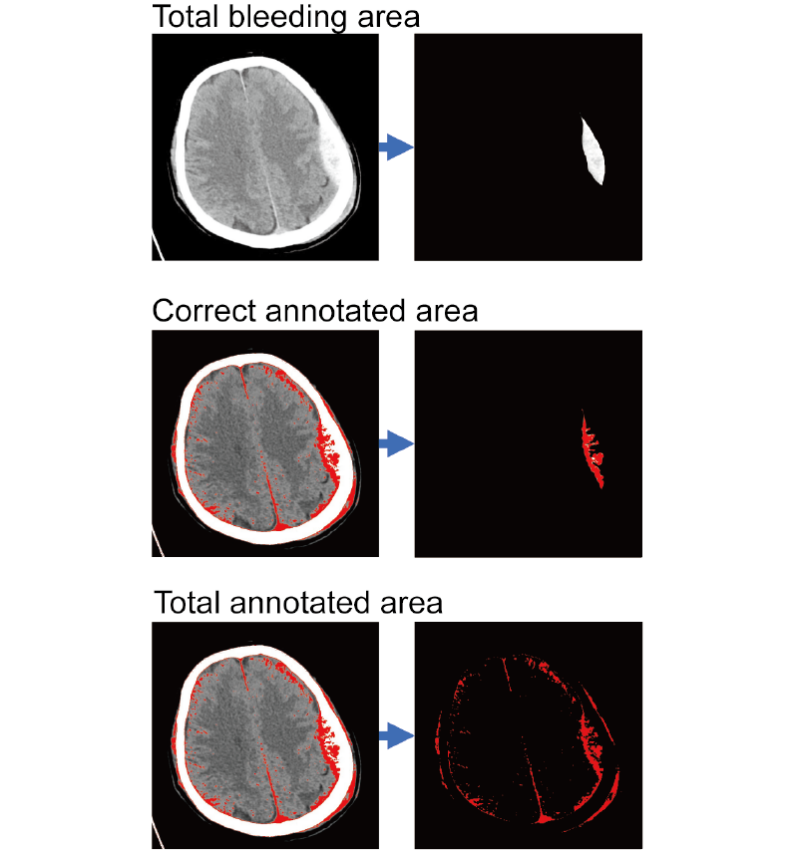
Calculation methods of identification completeness and misidentification percentage using Photoshop. The 3 pairs of pictures show the method of using Photoshop to isolate the total bleeding area, the correct annotated area, and the total annotated area.

After the size of the exact image’s bleeding and annotated part were counted, the identification completeness and misidentification percentages were calculated through the following formulas (Figure 2):

Identification completeness percentage = correct annotated area / total bleeding area × 100%

Misidentification percentage = (total annotated area – correct annotated area) / total annotated area × 100%

### Radiologist Evaluation

For the purpose of evaluating the competence of GPT-4 in cerebral hemorrhage identification, 10 professional radiologists were invited to compare the original CT images and the annotated images. We designed a 4-point scale questionnaire. In this questionnaire, the original image and the annotated image were compared in pairs, and those pairs of CT images were shown to the radiologists. To avoid ambiguous and unclear outcomes, the choices of the questionnaire did not contain neutral responses. The questionnaire was completed by radiologists separately and alone. The criteria and groups used for rating are shown in Multimedia Appendix 2.

### Statistical Analysis

The data in this study were all continuous and conformed to normal distribution and homogeneity of variance; therefore, we chose the 2-tailed Student *t* test and 1-way ANOVA for statistical analysis. SPSS (version 16.0; IBM Corp) was used to perform all statistical analysis. Summary data are presented as mean and SD, with statistical significance assessed by 2-tailed Student *t* test for 2-group comparisons or 1-way ANOVA for comparisons with more than 2 groups. *P*<.05 was considered statistically significant.

## Results

A total of 208 CT scans of different types of cerebral hemorrhages were collected between January and September in 2023 from the radiology database. These images consisted of epidural hematomas (32 images); acute subdural hematomas (42 images); chronic subdural hematomas (30 images); subarachnoid hemorrhages (36 images); intraparenchymal hemorrhages (31 images); and complex hemorrhages, which included 2 or more different types of intracranial bleeding (37 images).

**Figure 3:**
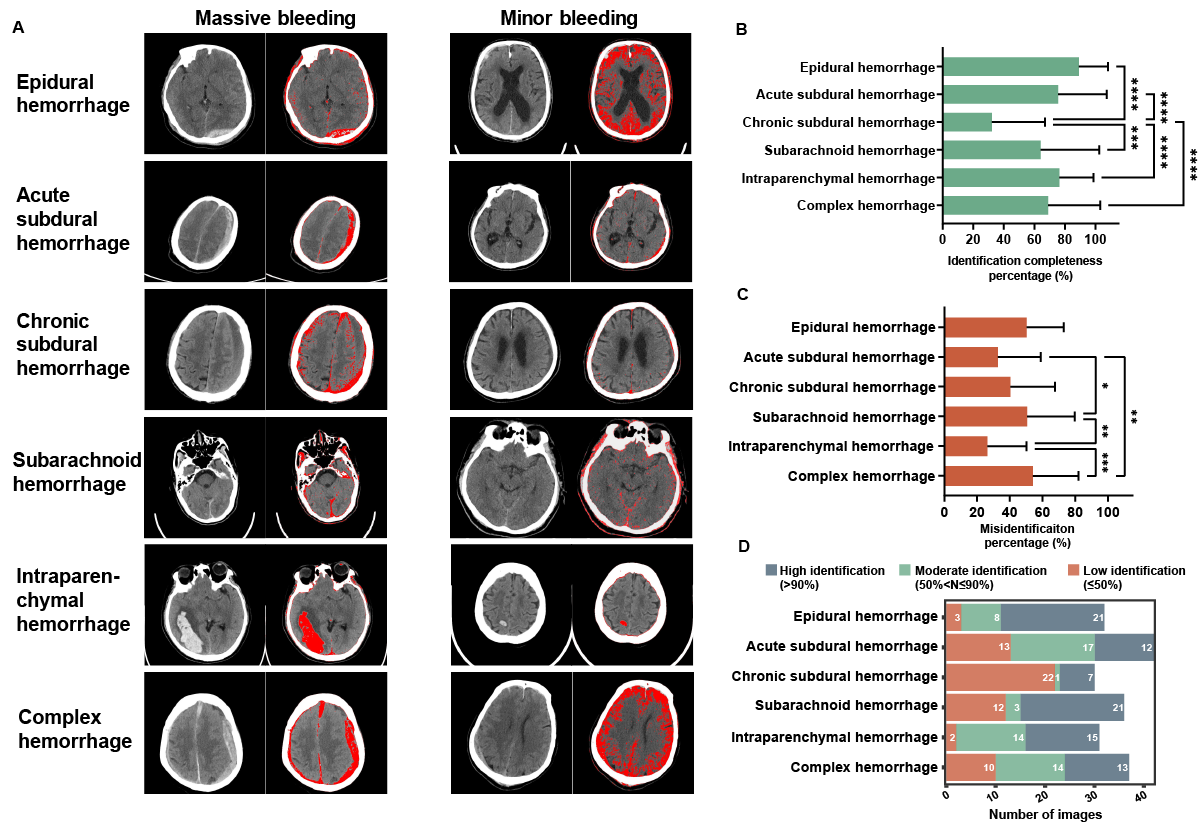
Identification completeness and misidentification in 6 types of cerebral hemorrhages. (A) Representative images of 6 types of cerebral hemorrhages, separated into the massive and minor bleeding groups using a benchmark of 5 mL for the volume of bleeding. (B and C) Identification completeness and misidentification percentage of different types of the cerebral hemorrhages (mean and SD); *P<.05, **P<.01, ***P<.001, and ****P<.0001 by 1-way ANOVA with Tukey multiple comparison test. (D) The distribution of different identification completeness percentage groups in 6 types of cerebral hemorrhages.

First and foremost, we were keen to assess whether GPT-4 could identify cerebral hemorrhages and the completeness of such detections. In Figure 3A, we present the performance of GPT-4 across 6 different types of hemorrhages, where red indicates the annotated hemorrhagic lesions identified by GPT-4. The overall identification completeness percentage for the 6 types of hemorrhages by GPT-4 reached 72.6% (SD 18.6%). Among these, GPT-4 demonstrated the highest identification completeness percentages for epidural and intraparenchymal hemorrhages, achieving 89.0% (SD 19.1%) and 86.9% (SD 17.7%), respectively. The identification completeness percentages for acute subdural hemorrhages (74.4%, SD 33.8%), subarachnoid hemorrhages (71.5%, SD 36.5%), and complex hemorrhages (76.4%, SD 32.9%) were also relatively high, whereas it was very low for chronic subdural hemorrhages (37.3%, SD 37.5%; Figure 3B). Nevertheless, the misidentification percentages for complex hemorrhages (54.0%, SD 28.0%), epidural hemorrhages (50.2%, SD 22.7%), and subarachnoid hemorrhages (50.5%, SD 29.2%) were high (>50%), whereas they were relatively low (<40%) for acute subdural hemorrhages (32.6%, SD 26.3%), chronic subdural hemorrhages (40.3%, SD 27.2%), and intraparenchymal hemorrhages (26.2%, SD 23.8%; Figure 3C). These results suggest that GPT-4 has a good capability to recognize some types of acute hemorrhages but lacks the ability to comprehensively identify chronic hemorrhages.

Further, we defined identification completeness percentages of >90%, from 50% to 90%, and ≤50% as high, moderate, and low identification, respectively. For epidural hemorrhages, the proportion of high identification reached 66% (21/32), and the proportion of moderate to high identification reached 91% (29/32), which further illustrated GPT-4’s strong capability in identifying epidural hemorrhages (Figure 3D). For intraparenchymal hemorrhages, the proportion of high identification was 48% (15/31), and the proportion of moderate to high identification was 94% (29/31), indicating that GPT-4 also had a good ability to recognize intraparenchymal hemorrhages (Figure 3D). In the cases of acute subdural hemorrhages and complex hemorrhages, approximately one-third of each set of images were categorized into high (12/42, 29% and 13/37, 37%, respectively), moderate (17/42, 40% and 14/37, 38%), and low (13/42, 31% and 10/37, 27%) identification; for subarachnoid hemorrhages, there was a substantial polarization (high identification at 21/36, 58% and low identification at 12/36, 33%), suggesting that GPT-4’s recognition of these 3 types of hemorrhages was not stable (Figure 3D). Finally, in chronic subdural hemorrhages, the proportion of low identification reached up to 73% (22/30), further showing GPT-4’s challenges in accurately identifying chronic subdural hemorrhages (Figure 3D).

**Figure 4:**
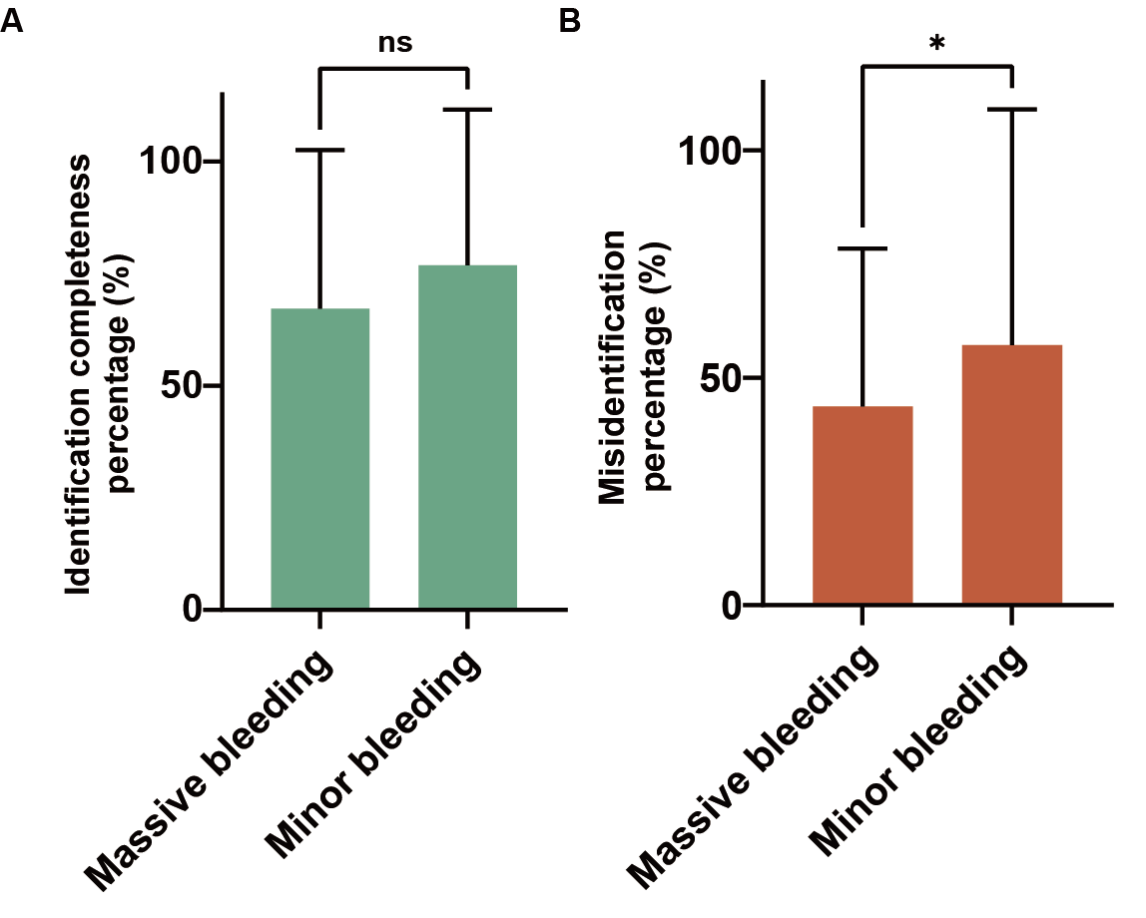
Identification completeness and misidentification percentage in the massive and minor bleeding groups. Cerebral hemorrhages were classified into massive and minor bleeding, using a benchmark of 5 mL for the volume of bleeding (mean and SD); *P<.05 and ns=*P*>.05 by 2-tailed t test. ns: no significance.

We classified cerebral hemorrhages into massive and minor bleeding, using a benchmark of 5 mL for the volume of bleeding. Surprisingly, there was no significant difference in the identification completeness percentages by GPT-4 between massive and minor bleeding (*P*=.06), indicating that the bleeding volume, within a certain range, did not affect GPT-4’s ability to identify cerebral hemorrhages (Figure 4A). However, the misidentification percentage was higher for minor bleeding than for massive bleeding, suggesting that GPT-4 was more adept at recognizing substantial cerebral hemorrhages (*P*=.04; Figure 4B).

**Figure 5:**
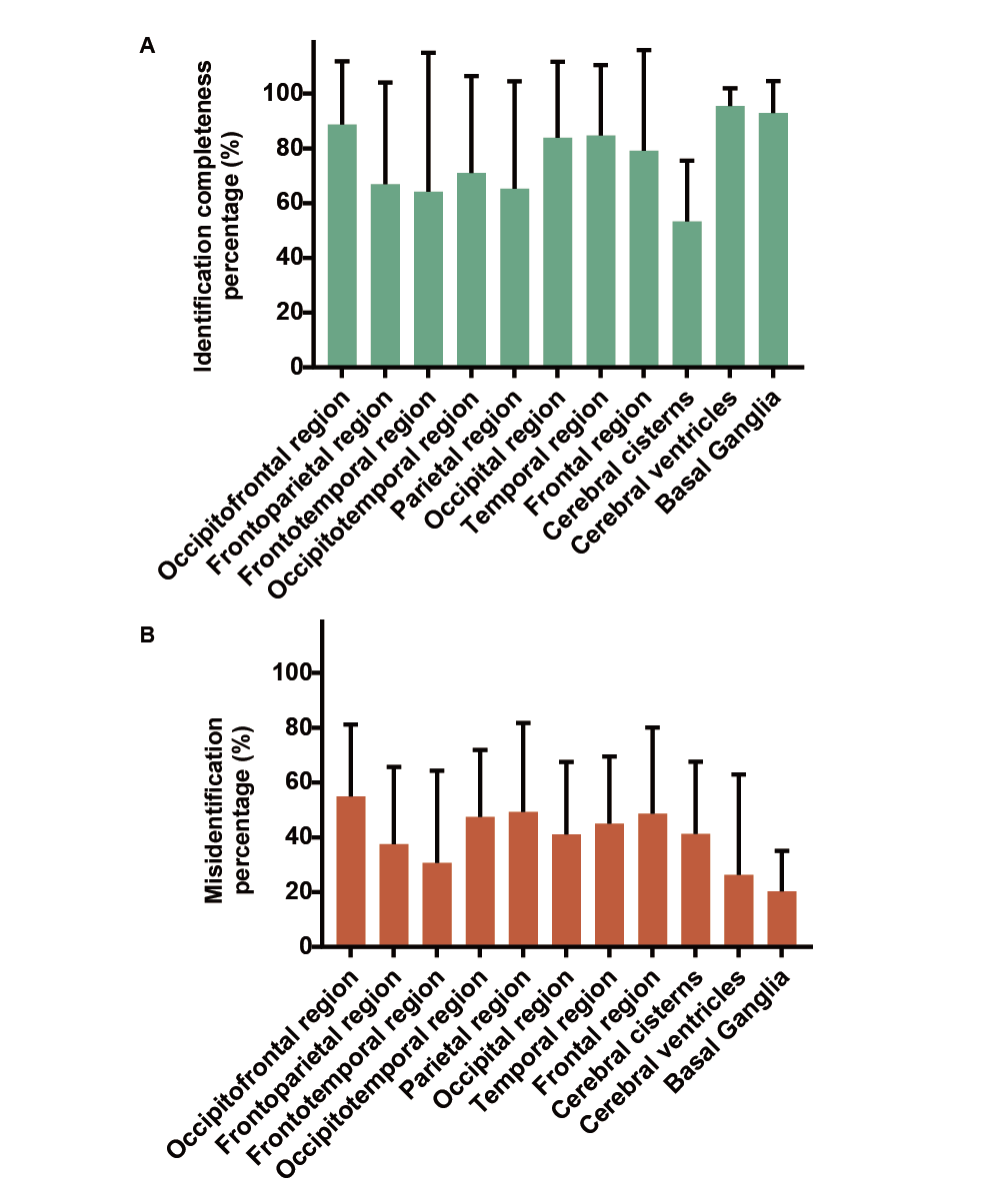
Identification completeness and misidentification percentage in the different locations of hemorrhage within the brain. Cerebral hemorrhages were classified according to different hemorrhagic locations (mean and SD); by 1-way ANOVA with Tukey multiple comparison test.

When we further performed analysis based on the location of hemorrhage within the brain, it was observed that GPT-4 demonstrated relatively higher identification completeness percentages and lower misidentification percentages in identifying hemorrhages in the cerebral ventricles and basal ganglia (Figure 5A and B). Conversely, the identification completeness percentage was comparatively lower for recognizing hemorrhages in the cerebral cisterns, and there was a higher misidentification percentage for hemorrhages in the occipitofrontal regions (Figure 5A and B). However, overall, there were no significant differences in the identification completeness percentage and misidentification percentage among different locations (all *P*>.05; Figure 5A and B). This indicated that GPT-4 did not show a marked preference for hemorrhages in specific areas but was relatively more proficient at identifying hemorrhages within the cerebral ventricles and basal ganglia.

**Figure 6:**
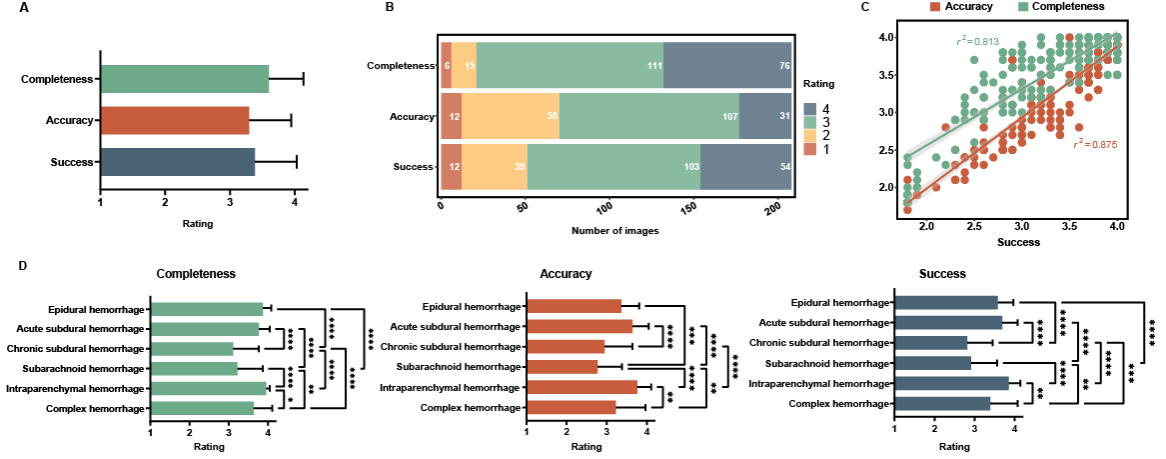
Evaluation on the CT identification by 10 radiologists. (A) The rating of the identification completeness, accuracy, and success (mean and SD). (B) The distribution of rating from identification completeness, accuracy and success. (C) The correlation between the identification completeness (green) or accuracy (red) and success. (D) The rating of identification completeness, accuracy, and success in 6 types of cerebral hemorrhages (mean and SD); *P<.05, **P<.01, ***P<.001, and ****P<.0001 by 1-way ANOVA with Tukey multiple comparison test. CT: computed tomography.

Based on the aforementioned results, we examined how radiologists viewed the performance of GPT-4 in identifying cerebral hemorrhages. Overall, radiologists expressed relative acceptance in terms of identification completeness (3.60, SD 0.54), accuracy (3.30, SD 0.65), and success ((3.38, SD 0.64; Figure 6A). Specifically, 89.9% (187/208) of CT images were accepted in terms of identification completeness; 66.3% (138/208) of CT images were accepted in terms of identification accuracy; and 75.5% (157/208) of CT images were accepted in terms of identification success (Figure 6B). Moreover, at the same level of identification success, the average rating for identification completeness was higher than that for identification accuracy, indicating that radiologists placed more importance on GPT-4’s ability to fully identify hemorrhagic lesions and relative tolerance for false positives (Figure 6C). Generally, whether it was in terms of identification completeness, accuracy, or success, the majority of CT scans were only deemed “relatively acceptable” (from 103/208, 49.5% to 111/208, 53.4%; Figure 6B). However, the proportion categorized as “completely unacceptable” was also very low (from 31/208, 14.9% to 76/208, 36.5%; Figure 6B). Further analysis was conducted based on different types of hemorrhages. The identification completeness, accuracy, and success of intraparenchymal hemorrhages and acute subdural hemorrhages received the highest acceptance from radiologists. Chronic subdural hemorrhages and subarachnoid hemorrhages had the lowest level of acceptance, with the identification completeness nearly reaching “relatively unacceptable” and both the identification accuracy and success being rated as “relatively unacceptable” (Figure 6D).

## Discussion

In this study, we explored the possibility of applying GPT-4 to identify cerebral hemorrhages directly from cranial CT images, implying that NLP models like GPT-4 could be of clinical and commercial value in practice.

Overall, GPT-4 can identify most cerebral hemorrhage CT images after simple language-based training, achieving an overall identification completeness percentage of 72.6% (SD 18.6%). If chronic subdural hemorrhage is not considered, then the identification completeness percentage improves to 79.6% (SD 7.8%). This is significantly higher than our initial expectations prior to conducting this study, and radiologists have expressed an acceptable attitude toward this outcome (Figure 6A).

Specifically, in terms of different types of cerebral hemorrhages, GPT-4 demonstrates relatively high identification completeness percentages for epidural and intraparenchymal hemorrhages, with 89.0% (SD 19.1%) and 86.9% (SD 17.7%), respectively (Figure 3B). However, its identification completeness percentage for chronic subdural hemorrhage is very low, at only 37.3% (SD 37.5%; Figure 3B). The reason for this is that acute hematomas have a higher CT value compared to normal brain tissue, making the hematoma on CT images appear “whiter,” thus easier for GPT-4 to differentiate from normal brain tissue. In contrast, chronic hematomas have CT values closer to that of normal brain tissue, making it challenging for GPT-4 to identify them based on image brightness variations. Radiologists diagnose chronic subdural hematomas more on the basis of changes in brain tissue symmetry, hematoma shape, and the absence of brain sulci and gyri structures in the hematoma, rather than just changes in CT values [31]. These are aspects that the current version of GPT-4 struggles to comprehend. Additionally, this may also be related to the inadequacy of the prompts provided to GPT-4. We attempted to instruct GPT-4 to recognize changes in the symmetry of brain tissue structures, but this resulted in even poorer identification completeness for chronic subdural hemorrhage (data not included). The current version of GPT-4 may not yet fully grasp human anatomical structures and their alterations. This suggests that if NLP models are to be applied in clinical scenarios, they may need enhanced training in fundamental medical knowledge.

In terms of identification accuracy, GPT-4’s performance was not consistent. Compared to minor bleeding, GPT-4 was relatively more accurate in identifying massive bleeding (Figure 4B). This might be because it is challenging for GPT-4 to distinguish smaller hematomas from normal brain tissue due to their limited size. GPT-4 demonstrated relatively greater precision in recognizing hematomas in the cerebral ventricles and basal ganglia, possibly because these hematomas were located in the central part of the cranial CT scans, where their grayscale values were less likely to be negatively affected by the skull bones (Figure 5B). Therefore, for larger and relatively isolated hemorrhagic lesions, GPT-4 can identify them with more accuracy.

However, the current iteration of GPT-4 is not yet ready for clinical application and has the following limitations. First, the identification completeness and accuracy for some types of intracranial hemorrhages are relatively low. This directly prevents GPT-4 from being applied in clinical settings. Second, at present, GPT-4 can only recognize a single CT image plane and cannot recognize a series of continuous CT images. The specific choice of which image plane to use still requires a doctor’s experience to decide. Third, a simple language-based training is needed before using GPT-4; therefore, inappropriate prompt words can greatly affect the recognition effectiveness of GPT-4. Fourth, the use of GPT-4 to identify intracranial hemorrhage also involves ethical and legal issues, which may not be universally accepted.

Nevertheless, we believe that the future trend will see AI assisting clinicians and enhancing efficiency in their work. Imagine scenarios like this: in hospitals lacking radiologists, assistants without medical background could upload patients’ head CT images to GPT-4 for preliminary screening; young radiologists could use GPT-4 to analyze cerebral hemorrhage CT images, helping to avoid missed diagnoses; and in extraordinary situations like wars, earthquakes, or maritime disasters, even laypeople without medical training would not be perplexed when facing head CT scans. From the evaluation of 10 radiologists, it appears that they are more concerned about GPT-4’s ability to identify hematomas, showing more tolerance toward misidentifications (Figure 6C). After all, for NLP models, not missing a hematoma holds greater clinical significance. In addition to clinical work, GPT-4 could potentially play a more significant role in medical education. In the traditional learning of CT or other radiologic imaging, medical students typically only receive images and the correct answers. Identifying the exact part of the image that represents the correct answer often leaves students confused. This approach focuses more on the result than the learning process. However, GPT-4 can assist in identifying abnormalities in images, and we can even envisage a future where GPT-4 could provide the correct diagnosis step by step. This shift would make medical education more process oriented and efficient.

In conclusion, this study represents the first exploration of applying GPT-4 to the identification of cerebral hemorrhages in cranial CT images, as well as the first attempt to utilize GPT-4 in the identification of radiological images. The current version of GPT-4 is capable of identifying a small portion of cerebral hemorrhages, indicating that it has a foundation for computer-assisted diagnosis. We believe that in the future, NLP models like GPT-4 will demonstrate immense clinical and commercial value.

## Appendix

Multimedia Appendix 1 Representative steps of computed tomography (CT) images inputted into GPT-4. (A) The initial natural language prompt is inputted with the CT image into GPT-4, assisting GPT-4 to inspect the original picture and give the annotation back. Users’ feedbacks help GPT-4 successfully annotate the bleeding area precisely. (B) The training CT image.

Multimedia Appendix 2 Criteria and categories used for rating of annotated images by radiologists.

## Abbreviations

AI: artificial intelligence
CT: computed tomography
NLP: natural language processing
