# Supplementary figures and images for: Using Natural Language Processing (GPT-4) for Computed Tomography Image Analysis of Cerebral Hemorrhages in Radiology: Retrospective Analysis
Source: J Med Internet Res. 2024 Sep 26;26:e58741. doi: 10.2196/58741 (PMC11467597; doi:10.2196/58741)

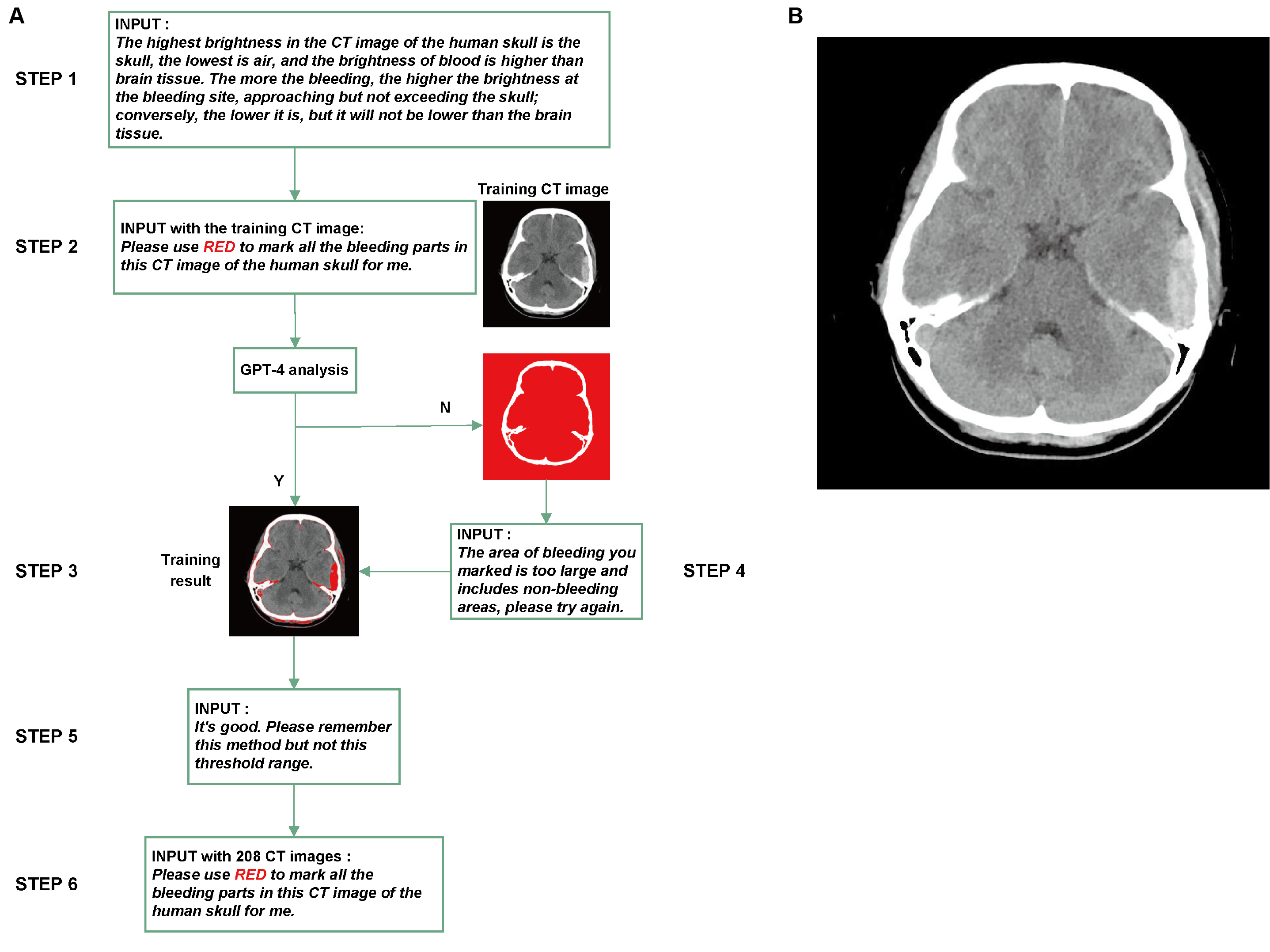

Supplement: Multimedia Appendix 1 [file jmir_v26i1e58741_app1.png]
