# Supplementary material for: Using Natural Language Processing (GPT-4) for Computed Tomography Image Analysis of Cerebral Hemorrhages in Radiology: Retrospective Analysis
Source: J Med Internet Res. 2024 Sep 26;26:e58741. doi: 10.2196/58741 (PMC11467597; doi:10.2196/58741)
Supplement: Multimedia Appendix 2 [file jmir_v26i1e58741_app2.docx]

**Multimedia Appendix 2: Criteria and Categories Used for Rating of Annotated Images by Radiologists**

| Criterion | Description |
| --- | --- |
| **Accuracy** | |
| 1: Completely unacceptable | Only a little bit of the hemorrhage is annotated by GPT-4. |
| 2: Relatively unacceptable | Less than half of the hemorrhage is correctly annotated by GPT-4. |
| 3: Relatively acceptable | Parts of the hemorrhage are mostly annotated by GPT-4, except for a few tiny bits. |
| 4: Completely acceptable | Parts of the hemorrhage are fully annotated by GPT-4 without any omission. |
| **Completeness** | |
| 1: Completely unacceptable | The annotated areas marked by GPT-4 consist mainly of non-bleeding areas, much more than hemorrhage areas. |
| 2: Relatively unacceptable | The annotated areas marked by GPT-4 are partially composed of non-bleeding areas, including parts with hemorrhage. |
| 3: Relatively acceptable | The annotated areas marked by GPT-4 are mainly the hemorrhage parts including a few non-bleeding areas. |
| 4: Completely acceptable | The annotated areas marked by GPT-4 are almost exactly the hemorrhage areas. |
| **Success** | |
| 1: Completely unacceptable | Both accuracy and completeness of the annotation by GPT-4 are totally disappointing. |
| 2: Relatively unacceptable | Neither accuracy nor completeness of the annotation by GPT-4 is sufficiently satisfactory. |
| 3: Relatively acceptable | GPT-4 has basically annotated the hemorrhage areas correctly without major false positives. |
| 4: Completely acceptable | Considering both the accuracy and completeness of the annotation, GPT-4 has precisely and fully annotated the hemorrhage areas. |
